# Effect of replacing concentrate diet with oat brewery waste on enteric methane emissions, nutrients intake, digestibility and compositional and functional abundances of rumen microbiota in growing male sheep

**DOI:** 10.3389/fmicb.2026.1646477

**Published:** 2026-01-27

**Authors:** Vedant Jayeshkumar Prajapati, Archit Mohapatra, Shraddha Trivedi, Pradeep Kumar Malik, Atul Purushottam Kolte, Umaya R. Suganthi, Tsuma Victor, Elena Ahasic, Artabandhu Sahoo, Raghavendra Bhatta

**Affiliations:** 1 ICAR-National Institute of Animal Nutrition and Physiology, Bangalore, India; 2 ICAR-Indian Veterinary Research Institute, Izatnagar, India; 3 International Atomic Energy Agency, Vienna, Austria; 4 Indian Council of Agricultural Research, New Delhi, India

**Keywords:** enteric CH4, metatranscriptomics, oat brewery waste, rumen microbiota, sheep

## Abstract

A study was carried out to examine the impact of two levels of oat brewery waste (OBW) on enteric methane (CH4) emissions, feed intake, rumen fermentation, rumen microbiota, and growth performance. A total of 21 male *Bannur* sheep in their growth phase were randomly assigned to three groups using a completely randomized design, receiving diet containing three levels of OBW: 0% (control group; C), 20% (T_1_), and 30% (T_2_). To achieve these levels in the diet, the concentrate was accordingly replaced with OBW (w/w). The finger millet straw and concentrate were offered separately, maintaining the concentrate to roughage ratio of 50:50. Aflatoxin B1 had no presence in the fresh OBW. The dry matter intake (g/day) in T_2_ group was significantly lower (*P* < 0.05) as compared to C and T_1_ groups. Sheep in T_1_ and T_2_ groups showed significantly lower (*P* < 0.05) daily CH_4_ emissions, decreased by 14%−15.5% as well as lower total energy loss (*P* < 0.05) compared to the group C. The feeding of OBW resulted in a notable decrease (*P* < 0.05) in volatile fatty acid (VFA) concentration (mmol) in the group T_2_ as compared to C. A noteworthy reduction (*P* < 0.05) in the total counts of protozoa and *Entodinimorphs* was observed at the 30% OBW (T_2_). The findings indicated that feeding OBW did not negatively affect the growth performance of sheep. Metatranscriptomic data from 15 individual animals, 5 from each group indicated that Bacteroidota was the predominant phylum, making up approximately 40% of the rumen microbiota. Their compositional abundance showed a significant decrease with the feeding of OBW at specified levels in present study. Conversely, there was an increase in the compositional abundance of Pseudomonadota in the test groups. Though the feeding of oat brewery waste in short-term (90 days) did not restructure the archaeal community composition, nevertheless it significantly affects the metabolic capabilities of the methylotrophic methanogens. The findings indicated that the OBW at a level of 20% in the diet led to a significant reduction (*P* < 0.05) in daily enteric CH4 emissions while not affecting feed intake, digestibility, or growth performance. Replacing concentrate with OBW at a level of 20% of diet can lead to monetary benefits while also providing the added advantages of lowering CH4 emissions.

## Introduction

Methane (CH4) is the second most potent greenhouse gas after carbon dioxide in terms of concentration in the atmosphere, contributing to a 30% increase in global temperature (IEA, 2025). The atmospheric concentration of CH4 in January 2025 was measured at 1,940 parts per billion (ppb), representing an increase of nearly 107 ppb compared to the 1,833 ppb recorded in January 2015. Livestock through enteric fermentation and manure are accountable for 32% of total anthropogenic CH4 emissions (UNEP, 2023). Further, the CH4 emissions from livestock also leads to a loss of feed (Johnson and Johnson, 1995; Morgavi et al., 2023). An adult animal daily produce 250–500 L CH4 (Johnson and Johnson, 1995), and for each liter of CH4, there is an approximate loss of 55 megajoules energy. For the feeding of livestock, many regions in developed nations have surplus production of feedstuffs (Schnepf, 2011), and therefore can compensate the energy loss by additional allowances of feed or supplemental feeding (Ma et al., 2025). Nonetheless, minimizing the energy loss in CH4 present a significant challenge especially in the feed resource scarce countries. Various efforts have made to develop cost-effective strategies for mitigating CH4 emissions. One of the approach could be to decrease the number of unproductive livestock; however, due to religious taboos particularly in developing countries, it is very difficult to follow (Malik et al., 2015). A viable alternative to mitigate CH4 emissions is the use of concentrate (Veen, 2000; Martin et al., 2010). The food-feed competition and high cost of concentrate pose a hindrance for their use in developing countries. Considering these facts, it is imperative to expand the low cost CH4 mitigation technologies while utilizing the resources which do not have a food-feed competition.

Alteration in the rumen microbiota composition and functionality offers possibilities for the mitigation of enteric CH4 emissions by feeding of brewery waste. It has been reported that brewery waste contained high phenol content (Tišma et al., 2018), which affect the rumen microbiota, methanogenesis (Baruah et al., 2019) and accumulation of H_2_ (Romero et al., 2023). Further, brewery waste also contains high crude protein, ether extract and energy content and could be used as a potential feed for the feeding of ruminants. The high fiber content of brewery waste restricts its use as a sole component of the diet. The brewing industries generate around 40 million metric tons of waste (Tan et al., 2021; Oyedeji and Wu, 2023), which can lead to the significant health concerns and environmental damage if not managed appropriately. The waste generated from brewery has significant nutritional value (Westendorf and Wohlt, 2002; Malik et al., 2024) and potential to replace costly concentrate in the diet. Recent *in vitro* studies conducted by Malik et al. (2024) have established the anti- methanogenic potential of oat brewery waste (OBW). The results from *in vitro* studies revealed a decrease of 38%−52% in CH4 production with the graded levels of concentrate replacement with OBW. The significant reduction in CH4 production was achieved at the partial replacement of concentrate with OBW at 20% level of the diet and beyond, whereas there was no significant impact on CH4 production at the lower levels. Further, a significant alteration in the rumen microbial community, protozoa numbers and dry matter and organic matter digestibility due to the replacement concentrate with OBW was noticed. Recent reports also established that the inclusion of brewery waste at 20%−30% level in the diet replacing concentrate ingredients is most optimum to achieve the comparable growth performance and improve feed efficiency (Ali, 2022; Chelkapally et al., 2025). Beyond these levels, the brewers grain linearly increase the neutral detergent fiber in the diet and adversely affect the dry matter intake (Chelkapally et al., 2025).

Based on our previous *in vitro* studies (Malik et al., 2024), and the recent reports available in the public domain, two inclusion levels of OBW i.e., 20 and 30% replacing concentrate in equal proportions (w/w) in the diet were selected for investigating the impact on enteric CH4 emissions, feed fermentation, and rumen microbiota in growing male sheep. We hypothesized that replacing (w/w) expensive concentrate with OBW at 20 and 30% levels in the diet can significantly decrease enteric CH4 emissions by altering rumen fermentation and microbiota composition without any negative impact on nutrient intake, digestibility, and productive performance of the sheep.

## Materials and methods

### Ethical approval

*In vivo* studies involving growing sheep were conducted to investigate the impact of replacing concentrate with OBW on nutrient intake, utilization, enteric CH_4_ emissions, and the compositional and functional abundances of rumen microbiota, adhering to the ethical guidelines set forth by the institute for animal handling. Approval was secured from the Institute Animal Ethics Committee under reference number NIANP/IAEC/1/2024/6 for conducting experiments on sheep, and all procedures were executed in strict adherence to the established guidelines of the IAEC.

### Animal, management and feeding

**Figure 1:**
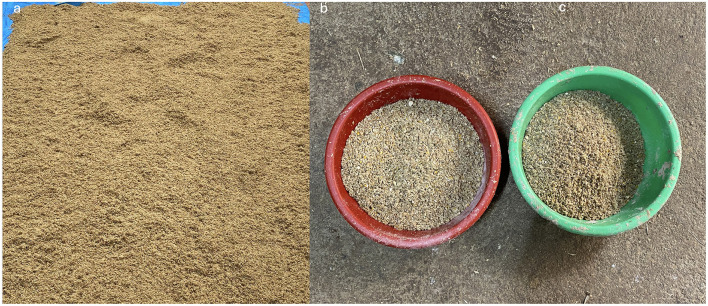
**(a)** Fresh oat brewery waste, **(b)** oat brewery waste mixed concentrate for feeding in group T_1_ and **(c)** oat brewery waste mixed concentrate for feeding of sheep in group T_2_.

Twenty-one *Bannur* male sheep, approximately 5 months old, having an average body weight of 17.7 ± 0.117 kg, were purchased from the Mandya district of Karnataka, India, and transported to the Experimental Livestock Farm of the National Institute of Animal Nutrition and Physiology in Bangalore (12°56′55″N 77°36′25″E). The growing sheep were randomly assigned into three groups of seven individuals each in a completely randomized design and accommodated in a well-ventilated shed arranged in a tail-to-tail configuration. Each sheep was assigned to an individual pen equipped for feeding and watering continuously over a 24-h period. The animals received deworming treatment with Ivermectin at a dosage of 200 mcg per kg of body weight. The growing sheep were fed according to the ICAR feeding standard (ICAR, 2013), ensuring their dry matter, energy, and protein needs were fulfilled. The diet consisted of equal parts finger millet (*Eleusine coracana*) straw and a concentrate. The concentrate was formulated by combining the ground components, including maize grain (320 g/kg), soybean meal (130 g/kg), groundnut cake (120 g/kg), wheat bran (400 g/kg), mineral mixture (20 g/kg), and salt (10 g/kg). In the control group (C), the sheep were offered a basal diet that consisted of equal parts of finger millet straw and concentrate, without OBW. In test group I (T_1_), the animals were offered a diet consisting of 50 parts finger millet straw, 30 parts concentrate, and the remaining 20 parts OBW. In test group II (T_2_), the animals offered 50 parts of finger millet straw, 20 parts of concentrate, and the remaining 30 parts comprised OBW. The concentrate without OBW (group C ) or with OBW (groups T_1_ and T_2_) was offered (Figure 1) in the morning at 09.00 h, whereas the finger millet straw was offered separately at 17.00 h. Daily the leftover of concentrate and finger millet straw was recorded and the intake was calculated. Based on the intake of individual animal and to ensure equal ratio of finger millet straw and concentrate, an additional 10% allowance was added to the daily offering of each so that the individual animal has *ad libitum* access to the feed. All the animals under different groups were adapted to the basal diet and local environment for 21 days before the onset of actual experiment.

The fresh OBW was collected each day from the SVP Farm in in Thattaguppe, Bangalore, India, and transported to the institute in the early morning hours. Three animal studies were carried out over 90 days to find out how replacing concentrate with OBW (w/w) at two different levels affected nutrient utilization, CH4 levels, and growth performance.

### Chemical composition, nutrient intake and digestibility

Every day, fresh OBW samples from the brewery were tested for aflatoxin B1 using an indirect competitive ELISA kit (PerkinElmer, Cat. No. SC0021) according to the instructions provided by the manufacturer. A total of 5 g of fresh OBW sample were placed in a 100 mL flask, followed by the addition of 25 mL of 60% methanol, and then vortexed for 5 min. Subsequently, the solution was placed into a tube and subjected to centrifugation at 4,000 × *g* for duration of 5 min. The supernatant underwent filtration using Whatman filter paper 1. Then, 1 mL of the filtered liquid was mixed with 4 mL of deionized water for 5 s, and 50 μL of this mixture was set aside for the ELISA test. The aflatoxin contamination in OBW was examined over a period of 3 days during aerobic storage in a well-ventilated covered shed.

The growing sheep underwent a 7-days metabolic trial after a 60-day preliminary feeding. Throughout the metabolic trial, the daily allowance of concentrate without (C) or with OBW (T_1_ and T_2_) as well as finger millet straw given to the individual sheep were recorded and the representative samples of feed offered were collected and dried in a hot air oven at 100°C for 24 h for the chemical composition analysis including energy content. The concentrate refusal for the individual sheep was recorded daily at 16.30 h, whereas the refusal of finger millet straw for the sheep individually was recorded next day at 08.30 h. The representative samples of refusals were collected and dried in a hot air oven at 100°C for 72 h. Based on the actual intake of concentrate and finger millet straw, the actual ratio of concentrate-roughage was calculated for the individual animal. The dung voided was daily collected throughout the metabolic trial in the fecal bags tied to the individual sheep, while the total urine was collected in a tightly screwed glass bottle placed under the metabolic cages and connected through a tube to the urine bags tied to the sheep. The total dung voided was weighed next day at 08.30 h for the individual sheep and the aliquots for dry matter (DM), N and energy estimation were collected, preserved and processed in accordance to Krishna and Ranjhan (1980). Daily, urine volume was measured and freeze dried for GE and N estimation (Morris et al., 2021). On the completion of metabolic trial, the dried samples were ground in a Cyclotec mill (CT293, FOSS, India) and stored for further analysis. The proximate components in the feed, refusals, and dung samples were analyzed in accordance with standard procedures. The crude protein (CP) was determined through the analysis of nitrogen content using the automatic nitrogen analyzer (VAPODEST 450, Gerhardt, Cäsariusstraße, Germany), followed by multiplication by 6.25. The ash content was determined through the incineration of a two-gram sample in a muffle furnace at 550°C for 4 h. In contrast, the organic matter (OM) was derived by calculating the difference between the dried weight of the sample and the ash content. The ether extract (EE) in the samples was determined according to AOAC (2005) utilizing a Soxtherm instrument (Gerhardt, Germany). The determination of fiber fractions in the samples, including neutral detergent fiber (NDF) and acid detergent fiber (ADF), was carried out following the methodology of Van Soest et al. (1991) using an automatic fiber analyzer (Fibertherm FT12, Gerhardt, Cäsariusstraße, Germany).

### CH_4_ measurement

Along with the digestibility study, the CH4 measurement was carried out in growing sheep using the sulfur hexafluoride (SF6) tracer technique (Berndt et al., 2014). In summary, the brass permeation tubes equipped with a Swagelok nut served as a source for the SF6 gas. The permeation tubes were filled with SF6 (99.9%) in liquid nitrogen and calibrated at 39°C by measuring tube weight weekly over a period of 12 weeks. The release rate (mg/day) was calculated using linear regression, taking into account the change in tube weight throughout the 12-week calibration period. Tubes with a *R*^2^ value of 0.99 or more were chosen for use in the experiment. Ten days before the CH4 measurement, the calibrated permeation tubes were placed in the rumen of sheep. The mean release rate of SF6 from the permeation tubes was 2.87 ± 0.128 mg/day. The vacuumed PVC canisters (>95 kPa) were fastened individually to collect the breath samples from sheep over a full feeding cycle of 24 h. Each day, the vacuumed PVC canisters were tied at 09:15 and removed next day from the animal after collecting the breath samples for the complete feeding cycle. Throughout the experiment, we obtained a minimum of five successful breath samples from each individual sheep. The N_2_-diluted breath gas samples were withdrawn using an airtight Hamilton glass syringe to analyze the sample in the gas chromatograph (GC 2010 plus, Shimadzu, Japan) equipped with a flame ionization detector (FID) and an electron capture detector (ECD) for the analysis of CH4 and SF6 gases, respectively. The similar conditions for gas chromatography were followed as described previously (Malik et al., 2021; Thirumalaisamy et al., 2022). The concentrations of CH4 (ppm) and SF6 (ppt) were determined following the methodology outlined by Lassey et al. (2014), with minor modifications for local elevation and atmospheric pressure.


$$\begin{array}{l} \left[G_{S}\right]=\frac{90-\tau_{f}}{\tau_{e}-\tau_{s}}x\left[G_{A}\right] \end{array}$$ (1)

G_S_ indicated the levels of CH4 (ppm) or SF6 (ppt) under atmospheric pressure of 90 kPa and at an elevation of 920 m. τ_*f*_ (kPa) indicated the final vacuum in the canister following N_2_ dilution, τ_*s*_ (kPa) denoted the post-sampling vacuum in the canister, while τ_*e*_ represented the vacuum in the evacuated canister. G_A_ referred to the concentration of CH4 (ppm) or SF6 (ppt) in the samples analyzed by the gas chromatograph.

Daily enteric CH4 emissions was calculated using the equation of Moate et al. (2014) and expressed as gram per day.


$$\begin{array}{l} R_{CH_{4}}=R_{SF_{6}}\frac{{\left[CH_{4}\right]}_{M}-{\left[CH_{4}\right]}_{BG}}{{\left[SF_{6}\right]}_{M}-{\left[SF_{6}\right]}_{BG}}\times \frac{MW_{CH_{4}}\;}{MW_{SF_{6}}}\times 1,000 \end{array}$$ (2)

*R*_CH4_ indicated the CH4 emission (g/day); *R*_SF6_ denoted the SF6 release rate from the tubes (mg/day); [CH4]_M_ – [CH44]_BG_ referred to the CH4 concentration in the sample and background; [SF6]_M_ – [SF6]_BG_ represented the SF6 concentration in the sample and background; MW_CH4_ and MW_SF6_ signified the molecular mass of CH4 and SF6, respectively.

The DM and OM intake, as well as their digestibility in the individual sheep during the experiment, were taken into consideration and the CH_4_ emissions was expressed as g/100 g DM intake, g/100 g OM intake, g/100 g digestible DM intake and g/100 g digestible OM intake.

### Energy partitioning

The gross energy (GE) of feed, refusals, fecal and urine samples was determined using a bomb calorimeter (RSB 7, Rajdhani Scientific Instrument, New Delhi, India). In brief, around 0.5 g of the ground dried sample was weighed and converted into pellets prior to being placed into the bomb crucible. The GE in urine sample was determined by drying 4 g of sample in a bomb capsule at 60°C (Morris et al., 2021). The GE was determined through the incineration of a sample in a sealed oxygen-rich environment, with the temperature raised during the combustion being recorded to ascertain the GE content. The GE was quantified in megajoules per kilogram of dry matter (MJ/kg DM). Partitioning of energy was determined according to Da Fonseca et al. (2019). In brief, the GE intake was calculated by multiplying DM intake with the GE of feed, whereas the energy lost through feces (FE) and urine (UE) was determined by taking the energy content of feces and urine into account and multiplying with the total quantity of dung and total volume of urine, respectively. Energy loss in CH4 was estimated by taking daily enteric CH4 emission and its energy value i.e., 55 MJ/L (World Nuclear Association, 2020) into account. The digestible energy (DE) was determined by subtracting the FE loss from the GE intake and expressed as MJ/day. Further, the metabolizable energy (ME) was calculated by subtracting the total UE and the energy lost through daily enteric CH4 emissions. The ME intake was expressed in MJ/day.

### Rumen fluid collection

At the completion of the trial, rumen fluid samples were collected 3 h after feeding from five sheep in each group selected randomly with the help of a stomach tube. About 45 mL of the rumen fluid, which included both fluid and solid components, was collected and equally divided into three subsets of 15 mL each. The first subset was set aside to extract metagenomic RNA, while the second and third subsets were used to measure ammonia-volatile fatty acid (VFA) and count protozoa, respectively. In the first subset for RNA isolation, the rumen digesta, which had both liquid and solid parts, was used without filtering it. The liquid part from the second subset, which was separated by spinning at 11,300 × *g* and 4°C for 15 min, was kept for determining ammonia-nitrogen (N) and VFA. To preserve the supernatant for determining ammonia-N, 2–3 drops of saturated HgCl_2_ were added, and for estimating VFA, 25% metaphosphoric acid was mixed and stored at −20°C until processing. The third subset of the digesta was used for the enumeration of ruminal protozoa.

### Fermentation characters

The preserved supernatant samples were thawed at room temperature for the determination of ammonia-N and VFA. The concentrations of individual VFAs were estimated according to the methodology outlined by Filípek and Dvorák (2009) using a gas chromatograph (Agilent 7890B, Santa Clara, USA) with the functional conditions described previously (Malik et al., 2021; Thirumalaisamy et al., 2022). The concentration of ammonia-N in the supernatant was measured following the method of Conway (1957). In brief, 1 mL of boric acid was placed into the inner chamber, while an equivalent volume of Na_2_CO3 was added to the outer chamber of the Conway disk. Subsequently, 1 mL of supernatant was put in the outer chamber opposite from the Na_2_CO3, and the disk was covered with a lid and allowed to remain undisturbed at room temperature for 2 h, after which titration was performed using 0.01 N H_2_SO4. The concentration of ammonia-N was determined using the following formula and expressed in mg/dL.


$$\begin{array}{l} \text{Ammonia}-N\;\left(\text{mg}/\text{dL}\right)=0.01H2SO4\text{ used in mL x }14N \end{array}$$ (3)

### Protozoal enumeration

The third subset of the rumen fluid collected from sheep was used for protozoal enumeration by combining equal volumes of the rumen fluid with formal saline. The mixed samples were allowed to remain undisturbed at room temperature overnight. The morphological characterization of protozoa was carried out following the methods outlined by Hungate (1966), while enumeration was performed using a phase-contrast microscope (Nikon Eclipse, Japan) at a 10 × objective, in accordance with the procedures of Kamra and Agarwal (2003).

### Growth study

Throughout the experimental period, the body weight of each sheep was recorded on a weekly basis. Feed intake was recorded daily, and the feed allowance was adjusted weekly according to body weight. The average daily gain (ADG, g/day) for individual sheep across all groups was determined by calculating the difference between final and initial body weights over a 90-day period, and to obtain the ADG, the result was divided by the total number of days. The ADG was expressed as g per day. The Feed conversion ratio (FCR) was calculated considering total feed consumed (kg, DM basis) by the individual sheep and dividing by the body weight gain (kg) of the individual animal over the entire period. The mean FCR in different groups was calculated based on the FCR of all seven sheep within the group.

### RNA isolation and metatranscriptome sequencing

To isolate RNA, the rumen digesta was first pelleted at a low speed of 10,000 × *g* for 5 min, then resuspended in 100 μL of RNAlater stabilizing solution (GCC Biotech, India) and preserved until further processing (Comtet-Marre et al., 2017). The RNA was isolated from 0.25 g preserved digesta sample using the RNeasy PowerMicrobiome Kit (Qiagen). All procedures were carefully followed in accordance with the manufacturer's instructions. The purity and concentration of RNA were determined using the Qubit 4.0 fluorometer (Invitrogen, USA) with the Qubit RNA BR Assay Kit (ThermoFisher Scientific, USA). RNA samples were sent for metatranscriptome sequencing using the NovaSeq 6000 (Illumina Inc., USA) to an external facility (Eurofin Genomics India Pvt. Ltd, Bangalore, India). The paired-end sequencing libraries were generated employing the Illumina TruSeq RNA library preparation kit, and the ligated products undergo size selection using AMPureXP beads. The product was amplified using the index primer according to the instructions provided in the kit protocol. The libraries enriched through PCR were subjected to purification using AMPureXP beads and were subsequently analyzed on the Agilent TapeStation 4200. The paired-end libraries were prepared for loading onto the Illumina platform to facilitate cluster generation and sequencing.

The raw metatranscriptome data was used for exploring the compositional abundances and functional analysis. The rumen metatranscriptome data containing total RNA obtained after quality filtration and removal of host contamination, were used for assessing the compositional abundances. In brief, the raw metatranscriptome data were processed to remove adapter contamination, filter low-quality reads, and correct misrepresented bases using Fastp v 0.23.2 (Chen, 2023). Subsequently, removal of the host contamination from the reads was performed using Hisat2 v2.2.1 (Kim et al., 2019), and the host-contaminated reads were mapped against the sheep genome Oar_v4.0 (RefSeq assembly accession: GCF_000298735.2), the unmapped read files were considered as cleaned reads. The processed cleaned paired and single-end reads were uploaded at BV-BRC version 3.30.19 for the microbial taxonomic affiliation by K-mer using Kraken2 database (Wood et al., 2019). The Kraken2 output files were exported to the Pavian v 0.8.4 (Breitwieser and Salzberg, 2020) to obtain microbial abundances at different taxonomic levels. The metatranscriptome data at different taxonomic levels obtained from the Pavian were normalized by Total Sum Scaling (TSS) and count filters at a cutoff value of minimum four reads with 80% prevalence in MicrobiomeAnalyst 2.0 (Lu et al., 2023).

For functional analysis, from the clean reads, the rRNA was removed using SortMeRNA (v4.3.6, Kopylova et al., 2012). The contigs were built by using rRNA depleted reads in MEGAHIT (v1.2.9; Li et al., 2015) at a length cutoff of 300 bp. The contigs were then used to predict the potential protein coding regions in Prodigal (v2.6.3, Hyatt et al., 2010). Thereafter, the non-redundant open reading frame (ORF) Catalog was prepared by clustering the amino acid sequences at an identity threshold of 95% using CD-HIT (v4.8.1, Li and Godzik, 2006). The non-redundant amino acid sequences were assigned to the KEGG orthologs (KO) and clusters of orthologous genes (COG) in GHOSTX (Kanehisa et al., 2017) and COG classifier (v2.0.0, Shimoyama, 2022; Galperin et al., 2021), respectively. The coverage of predicted unigene ORFs was determined using RNASeq tool in the CLC genomics workbench (v21.0.2; Qiagen, USA) and the transcripts per million (TPM) were added to the corresponding KOs and COG.

### Statistical analysis

Data was evaluated for the outliers using the identify outliers feature in GraphPad Prism version 10.4.0. The outliers were identified by using the animal traits data such as intake, digestibility, growth, fermentation and energy partitioning were evaluated for gaussian distribution through the Kolmogorov–Smirnov test at a 5% significance level, using GraphPad Prism version 10.4.0 (GraphPad Software, San Diego, CA, USA). The analysis of the data was carried out using a One-way ANOVA within the specified model.


$$\begin{array}{l} Y_{ij}=\;u+\tau_{i}+\;\epsilon_{ij} \end{array}$$ (4)

Where *Y*_*ij*_ represents the *j*^th^ observation (*j* = 1, 2,… 0.0.7) on the *i*^th^ group (*i* = 1, 2, 3). μ was the common effect of the experiment, τ_*i*_ represents the *i*^th^ group effect, and ∑_*ij*_ represents the random error due to the *j*^th^ observation of the *i*^th^ group. The mean of each group was compared with the mean of every other group. The significance between the groups was ascertained using Tukey *post-hoc* analysis at a family-wise alpha threshold and confidence level of 0.05 in GraphPad prism version 10.4.0.

The significance among the groups was established using non-parametric Kruskal–Wallis rank sum tests, followed by the Dunn *post-hoc* test (Miles et al., 2022) in R Studio. The alpha diversity of the metatranscriptome data was determined using the Shannon index, while the beta diversity was estimated using the Bray-Curtis dissimilarity index.

The differential expression in the functionality of rumen microbiota between the groups was checked by DESeq2 (Love et al., 2014) and the significance was ascertained at a FDR value cutoff of 0.05.

## Results

### Composition, nutrient intake and digestibility

Chemical composition (g/kg) of feed constituents such as finger millet straw, concentrate, OBW and diet is presented in Table 1.

**Table 1:** Chemical composition (g/kg DM) of feed ingredients and diet.

**Attributes**	**Straw**	**Concentrate**			OBW^*^	**Diet**		
		**C**	**T**_1_	**T**_2_		**C**	**T**_1_	**T**_2_
OM	938	923	938	946	972	933	940	941
CP	37.1	201	218	228	285	129	139	145
NDF	743	261	415	519	760	526	580	622
ADF	497	105	164	184	306	309	335	349
EE	11.5	19.7	27.7	31.5	43.4	17.0	21.1	27.0
Ash	61.8	77.1	61.8	53.6	28.4	67.1	59.7	58.6
GE (MJ/kg)	13.8	17.0	17.6	17.7	19.2	12.8	13.1	11.5

OBW, oat brewery waste; OM, organic matter; CP, crude protein (N × 6.25); NDF, neutral detergent fiber; ADF, acid detergent fiber; EE, ether extract; GE, gross energy; g/kg DM, gram per kilogram of dry matter. C, control group (without replacement of concentrate with oat brewery waste); T_1_, test group 1 where oat brewery waste replaced the equal proportion of concentrate (w/w) and constituted 20% of the total diet; T_2_, test group 2 where oat brewery waste was replaced with the equal proportion of concentrate (w/w) and finally constituted 30% of the total diet. Each value is based on six observations. ^*^Aflatoxin B1 was not detected in the OBW at the time of collection, however, the traces (0.20–0.45 ppb) were reported during the 3 days of storage.

Results from the digestibility trial indicated a significantly (*P* < 0.05) lower DM intake (g/day) in group T_2_ as compared to C and T_1_ (Table 2). Despite the similar DM intake, there was a significant difference (*P* < 0.05) in the digestible DM intake (g/day) between C and T_1_ groups. The actual ratio of concentrate-roughage in DM intake deviated from the ratio of offering (50:50) and it was recorded 65:35, 63:37, and 63:37 in C, T_1_ and T_2_ groups, respectively. The organic matter intake (g/day) and digestible organic matter intake (g/day) in the growing sheep was significantly (*P* < 0.05) lower in T_2_ as compared to C and T_1_ groups. The CP intake (g/day) was significantly (*P* < 0.05) different between the group T_1_ and T_2_, whereas the CP intake between C and T_1_, as well as C and T_2_, was comparable. In contrary, the digestible CP intake (g/day) was significantly (*P* < 0.05) higher in T_2_ as compared to C, while there was no significant (*P* > 0.05) difference between group C and T_1_. The NDF intake was significantly (*P* < 0.05) higher in group T_1_ as compared to group C, whereas the ADF intake in T_1_ was significantly (*P* < 0.05) than that was in group C and T_2_. The adjustment of NDF and ADF intake data to the corresponding digestibility revealed the significantly (*P* < 0.05) lower intake in group T_2_ (Figure 1).

**Table 2:** Effect of replacing concentrate with oat brewery waste in diet on nutrients intake and digestibility in sheep.

**Attributes**	**Groups**			**SEM**	***P***
	**C**	**T**_1_	**T**_2_		
DM intake^*^ (g/day)	801^b^	796^b^	696^a^	34.197	< 0.0001
Dig. DM intake (g/day)	524^a^	477^b^	381^c^	42.080	< 0.0001
OM intake (g/day)	747^b^	748^b^	655^a^	30.834	< 0.0001
Dig. OM intake (g/day)	519^c^	467^b^	382^a^	39.929	< 0.0001
CP intake (g/day)	103^ab^	111^b^	101^a^	3.055	0.0061
Dig. CP intake (g/day)	75.6^b^	74.2^b^	65.2^a^	3.258	< 0.0001
NDF intake (g/day)	421^a^	461^b^	433^ab^	11.850	0.0100
Dig NDF intake (g/day)	247^b^	232^b^	209^a^	11.050	< 0.0001
ADF intake (g/day)	247^a^	266^b^	243^a^	7.094	0.0045
Dig. ADF intake (g/day)	129^c^	117^b^	97.8^a^	9.086	< 0.0001

^*^The actual ratio of concentrate-roughage in DM intake was 65:35, 63:37 and 63:37 in C, T_1_ and T_2_ groups, respectively. SEM, standard error of mean; *P*, denotes the significant at 5% level. DM, dry matter; Dig. DM, digestible dry matter; OM, organic matter; Dig. OM, digestible organic matter; CP, crude protein; Dig. CP, digestible crude protein; NDF, neutral detergent fiber; Dig. NDF, digestible neutral detergent fiber; ADF, acid detergent fiber; Dig ADF, digestible acid detergent fiber; C, control group (no replacement of concentrate with oat brewery waste); T_1_, test group 1 where oat brewery waste replaced the equal proportion of concentrate (w/w) and constituted 20% of the total diet; T_2_, test group 2 where oat brewery waste replaced the equal proportion of concentrate (w/w) and constituted 30% of the total diet. Mean values bearing superscript (a, b) in a row differ significantly at *p* < 0.05.

### CH_4_ emissions

**Figure 2:**
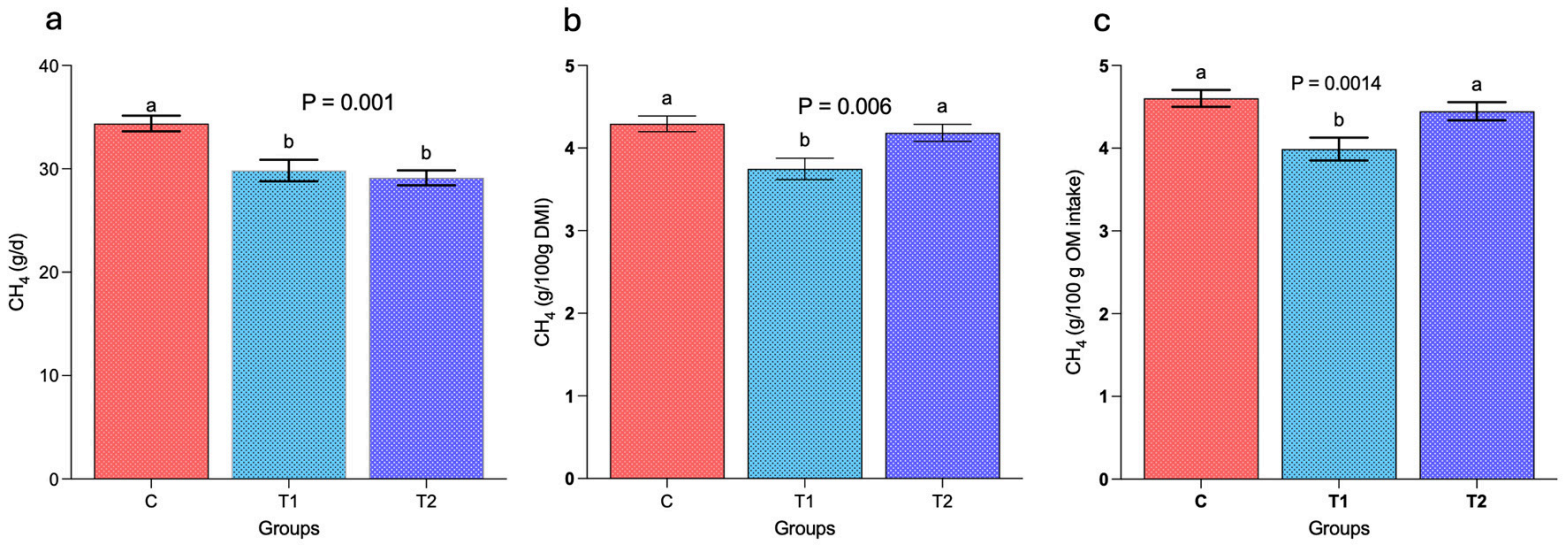
Effect of selected levels of oat brewery waste (OBW) on enteric CH_4_ emissions in growing sheep. **(a)** represents the daily CH_4_ emissions (g/day), whereas **(b, c)** represent the CH_4_ emissions, g per 100 g of dry matter intake (DMI) and per 100 g of organic matter (OM) intake. C, control group (no replacement of concentrate with oat brewery waste); T_1_, test group 1 where oat brewery waste replaced the equal proportion of concentrate (w/w) and constituted 20% of the total diet; T_2_, test group 2 where oat brewery waste replaced the equal proportion of concentrate (w/w) and constituted 30% of the total diet. Superscripts a, b on the top of the bar represents the significant at 5% confidence level and the mean values of the bar having different superscript differ significantly in CH_4_ emissions.

Data showed a significant (*P* < 0.05) decrease in daily enteric CH4 emissions (g/day) in group T_1_ and T_2_ as compared to group C. The replacement of concentrate with OBW at 20 and 30% of diet resulted into a significant (*P* < 0.05) decrease of 13.4 and 15.4% in daily enteric CH4 emissions in T_1_ and T_2_ groups, respectively (Figure 2a). These findings indicated that the replacement of concentrate with OBW beyond 20% of diet did not lead to any further significant (*P* > 0.05) decrease in daily enteric CH4 emissions (g/day). Adjustment of CH4 emissions data to per 100 g of DM intake in growing sheep indicated a significant (*P* < 0.05) decrease in CH4 emissions in T_1_ as compared to group C and T_2_ (Figure 2b). However, the CH4 emissions between C and T_2_ did not reveal any significant (*P* > 0.05) difference (4.29 vs. 4.14 g/100 g DM intake). Adjustment of CH_4_ emission data to per 100 g of OM intake revealed a remarkable (*P* < 0.05) reduction of about 13.2% in CH4 emissions was recorded in T_1_ as compared to group C (Figure 2c). On the contrary, the CH4 emissions between group C and T_2_ were comparable (4.60 vs. 4.45 g/100 g OM intake).

**Figure 3:**
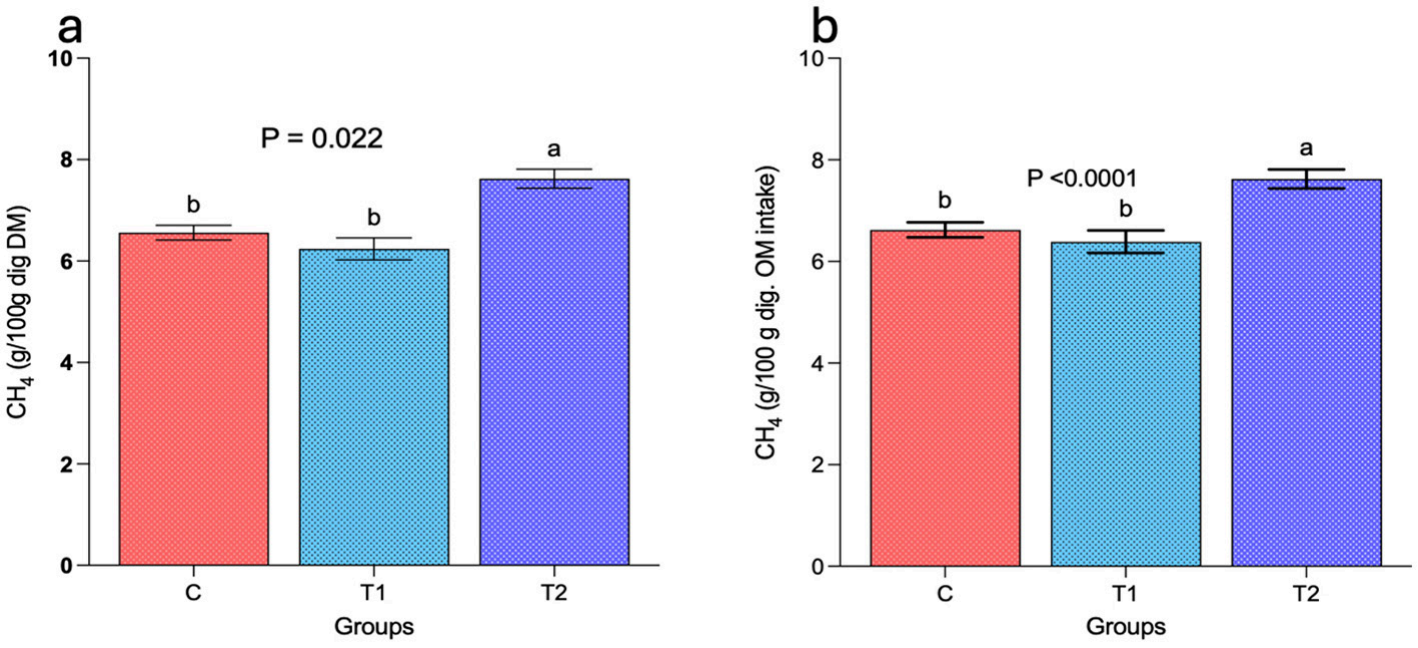
Effect of selected levels of oat brewery waste (OBW) on enteric CH_4_ emissions in growing sheep. **(a)** represents the CH_4_ emissions (g) per 100 g of digestible dry matter and **(b)** represents the CH_4_ emissions (g) per 100 g of digestible organic matter. C, control group (no replacement of concentrate with oat brewery waste); T_1_, test group 1 where oat brewery waste replaced the equal proportion of concentrate (w/w) and constituted 20% of the total diet; T_2_, test group 2 where oat brewery waste replaced the equal proportion of concentrate (w/w) and constituted 30% of the total diet.

Results from the study demonstrated significantly (*P* < 0.05) lower CH4 emissions in group C and T_1_ as compared to T_2_ when the emissions among the groups was compared based on per 100 g of digestible DM intake (Figure 3a) and per 100 g of digestible OM intake (Figure 3b). A reduction of about 18 and 16% in CH4 emissions was observed in group C and T_1_ as compared to T_2_.

### Energy partitioning

The GE intake (MJ/day) was higher (*P* < 0.05) in C and T_1_ groups as compared to T_2_ (Table 3). Similarly, the intake of DE was also significantly (*P* < 0.05) higher in C and T_1_ groups as compared to T_2_. The loss of energy in feces was significantly (*P* < 0.05) higher in group T_1_ as compared to C, whereas the fecal energy loss between C and T_2_ and T_1_ and T_2_ was comparable. However, the fecal energy loss (% of GE) was significantly (*P* < 0.05) higher in group T_2_ as compared to the other two groups. The fecal energy loss (% of GE) was minimum in group C (Table 3), however, this group had the maximum loss of GE in the form of CH4 (14.8 vs. 12.5 vs. 13.9 %). On the other hand, the UE (MJ/day) loss was lowest (*P* < 0.05) in group C as compared to groups T_1_ and T_2_. Overall, the ME intake (MJ/day) was similar in group C and T_1_, whereas sheep in T_2_ had the lowest (*P* < 0.05) ME intake.

**Table 3:** Effect of replacing concentrate with oat brewery waste in diet on energy partitioning in growing sheep.

**Attributes**	**Groups**			**SEM**	***P***
	**C**	**T**_1_	**T**_2_		
GE intake (MJ/day)	12.8^b^	13.1^b^	11.5^a^	0.491	< 0.0001
FE loss (MJ/day)	4.56^a^	5.51^b^	4.79^ab^	0.286	0.0498
FE loss (% of GE)	34.2^a^	40.2^b^	44.3^c^	2.932	< 0.0001
DE intake (MJ/day)	8.23^b^	7.64^b^	6.44^a^	0.527	< 0.0001
Energy loss in CH_4_ (MJ/day)	1.89^b^	1.64^b^	1.60^b^	0.091	0.0001
CH_4_ energy (% of GE)	14.8^b^	12.5^a^	13.9^b^	0.669	0.0003
UE loss (MJ/day)	0.201^a^	0.363^b^	0.439^b^	0.070	0.0003
UE (% of GE)	1.58^a^	2.81^b^	3.79^b^	0.639	< 0.0001
Total energy loss (%)	46.4^a^	52.9^b^	57.1^b^	3.112	0.0004
ME intake (MJ/day)	6.90^b^	6.17^b^	4.98^a^	0.560	< 0.0001

SEM, standard error of mean; *P*, denotes the significant at 5% level. GE, gross energy; DE, digestible energy; FE, fecal energy; CH_4_, methane; UE, urinary energy; ME, metabolizable energy; MJ/day, mega joule per day; C, control group (no replacement of concentrate with oat brewery waste); T_1_, test group 1 where oat brewery waste replaced the equal proportion of concentrate (w/w) and constituted 20% of the total diet; T_2_, test group 2 where oat brewery waste replaced the equal proportion of concentrate (w/w) and constituted 30% of the total diet. Mean values bearing superscript (a, b) in a row differ significantly at *p* < 0.05.

### Rumen fermentation

The ammonia-N (mg/dL) levels did not change (*P* > 0.05) when concentrate was replaced with two amounts of OBW in groups T_1_ and T_2_, and the levels were similar across the groups (Table 4). However, the total volatile fatty acid (TVFA) concentration (mmol) was significantly (*P* < 0.05) lower in group T_2_ compared to C and T_1_, whereas the TVFA concentration between C and T_1_ was not different (*P* > 0.05). Similarly, the acetate and propionate concentrations (mmol) were also recorded as the lowest in group T_2_. The concentration of other VFAs except isovalerate was not different (*P* > 0.05) among the groups (Table 4). These findings indicated that the replacement of concentrate with the OBW at the highest level, i.e., 30%, affected the VFA production adversely, whereas the replacement at the 20% level did not affect VFA production significantly (*P* > 0.05) compared to group C.

**Table 4:** Effect of replacing concentrate with oat brewery waste in diet on rumen fermentation.

**Attributes**	**Groups**			**SEM**	***P***
	**C**	**T**_1_	**T**_2_		
Ammonia-N (mg/dL)	29.1	25.5	22.9	1.797	0.1852
TVFA (mmol)	90.8^b^	84.1^ab^	73.0^a^	5.190	0.0088
Acetate (mmol)	58.9^b^	56.7^b^	49.2^a^	2.936	0.0027
Propionate (mmol)	17.6^b^	14.8^ab^	13.7^a^	1.160	0.0199
A/P Ratio	3.36	3.89	3.61	0.153	0.0899
Butyrate (mmol)	12.4	10.9	8.62	1.098	0.2656
Iso-butyrate (mmol)	0.836	0.702	0.684	0.058	0.3498
Valerate (mmol)	1.01	0.825	0.804	0.078	0.4688
Iso-valerate (mmol)	0.217^a^	0.080^b^	0.049^b^	0.058	0.0020

SEM, standard error of mean; *P*, denotes the significant at 5% level. TVFA, total volatile fatty acids; A/P, acetate to propionate ratio; g/day, gram per day; mg/dL, milligram per deca liter; mmol, millimole; C, control group (no replacement of concentrate with oat brewery waste); T_1_, test group 1 where oat brewery waste replaced the equal proportion of concentrate (w/w) and constituted 20% of the total diet; T_2_, test group 2 where oat brewery waste replaced the equal proportion of concentrate (w/w) in the diet and constituted 30% of the total diet. Mean values bearing superscript (a, b) in a row differ significantly at *p* < 0.05.

### Ruminal protozoa

**Figure 4:**
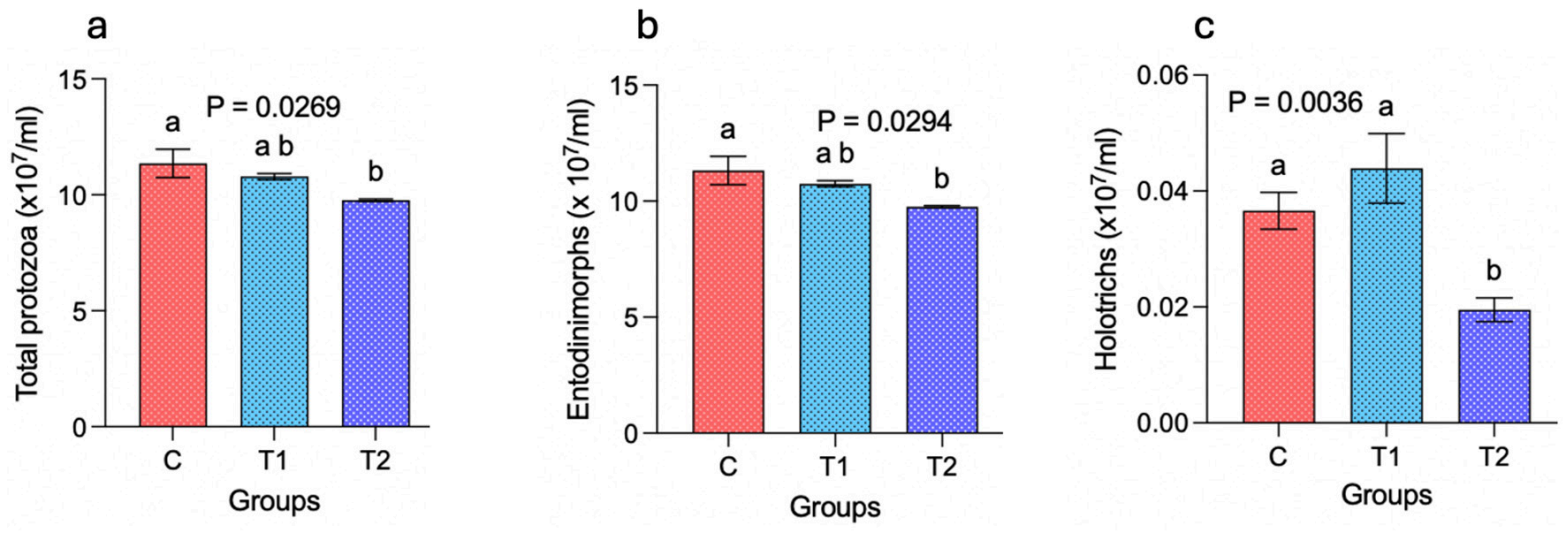
Effect of selected levels of oat brewery waste (OBW) on ruminal protozoa in growing sheep. **(a)** represents the total protozoa, whereas **(b, c)** represent the numbers of *Entodinimorphs* and *Holotrichs*, respectively. C, control group (no replacement of concentrate with oat brewery waste); T_1_, test group 1 where oat brewery waste replaced the equal proportion of concentrate (w/w) and constituted 20% of the total diet; T_2_, test group 2 where oat brewery waste replaced the equal proportion of concentrate (w/w) and constituted 30% of the total diet.

The total protozoa (cells × 10^7^/mL) were significantly (*P* < 0.05) decreased with the replacement of concentrate with OBW at the 30% level in T_2_ as compared to group C. However, the total protozoal numbers between C–T_1_ and T_1_-T_2_ were comparable (Figure 4a). Similarly, the numbers of *Entodinimorphs* (cells × 10^7^/mL) were decreased (*P* < 0.05) in T_2_ as compared to the control, but there was no difference in the *Entodinimorphs* numbers between the other groups (Figure 4b). The *Holotrichs* were significantly (*P* < 0.05) lower in group T_2_ as compared to groups C and T_1_ (Figure 4c).

### Growth

The initial and final body weight of the sheep showed no significant difference (*P* > 0.05) among the groups (Table 5). Similarly, the ADG (g) was also not significant (*P* > 0.05) between the C and test groups T_1_ and T_2_. There was a significant (*P* < 0.05) difference in the daily DMI (g/day) and total DMI for 90 days between the group T_1_ and T_2_. The FCR in C, T_1_ and T_2_ groups was 7.61, 6.87, and 6.77; however, there was no significant (*P* > 0.05) difference in the FCR among the groups.

**Table 5:** Effect of replacing concentrate with oat brewery waste in diet on growth performance in sheep.

**Attributes**	**Groups**			**SEM**	***P***
	**C**	**T**_1_	**T**_2_		
Initial BW (kg)	17.4	17.8	17.7	0.120	0.846
Final BW (kg)	25.6	26.7	26.1	0.318	0.257
ADG (g)	90.3	98.8	93.6	2.474	0.617
Mean DMI (g/day)	656^ab^	666^b^	622^a^	13.316	0.036
Total DMI (kg)	59.0^ab^	60.0^b^	56.0^a^	1.201	0.036
FCR	7.61	6.87	6.77	0.264	0.469

BW, body weight; ADG, average daily gain; DMI, dry matter intake; g/day, gram per day; kg, kilogram; SEM, standard error of mean; *P*, denotes the significance at 5% level. C, control group (no replacement of concentrate with oat brewery waste); T_1_, test group 1 where oat brewery waste replaced the equal proportion of concentrate (w/w) and constituted 20% of the total diet; T_2_, test group 2 where oat brewery waste replaced the equal proportion of concentrate (w/w) in the diet and constituted 30% of the total diet. Mean values bearing superscript (a, b) in a row differ significantly at *p* < 0.05.

### Compositional and functional abundances

**Figure 5:**
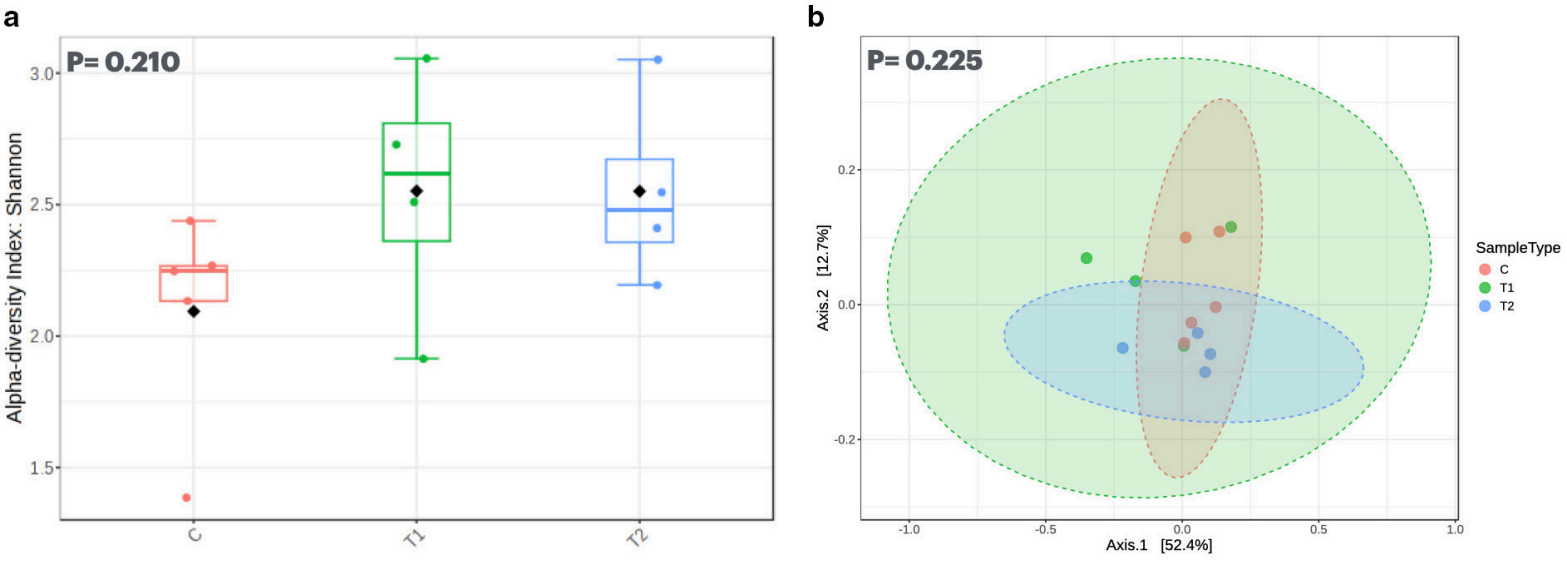
**(a)** represents the alpha diversity index of the rumen microbiota assessed through Shannon index method, whereas **(b)** represents the beta diversity based on the Bray-Curtis method. C, control group (no replacement of concentrate with oat brewery waste); T_1_, test group 1 where oat brewery waste replaced the equal proportion of concentrate (w/w) and constituted 20% of the total diet; T_2_, test group 2 where oat brewery waste replaced the equal proportion of concentrate (w/w) and constituted 30% of the total diet.

The alpha diversity measured with the Shannon index showed no significant difference (*P* > 0.05, Figure 5a), suggesting that the species richness and evenness across the groups were relatively consistent. Beta diversity (Figure 5b) analyzed the differences in microbial composition between the groups and showed no significant differences (*P* > 0.05). In this study, a total of 432 million transcriptomic raw reads were generated, with an average of 33.2 million per sample (Supplementary File 1). Approximately 4.32% of reads were excluded during quality filtration due to poor quality, short length (< 150 bp), and adapter contamination. Additionally, 0.145% of the metatranscriptomics reads were excluded because of host contamination. Subsequently, the high-quality paired and single-end metatranscriptomics reads were analyzed for taxonomical abundances.

**Figure 6:**
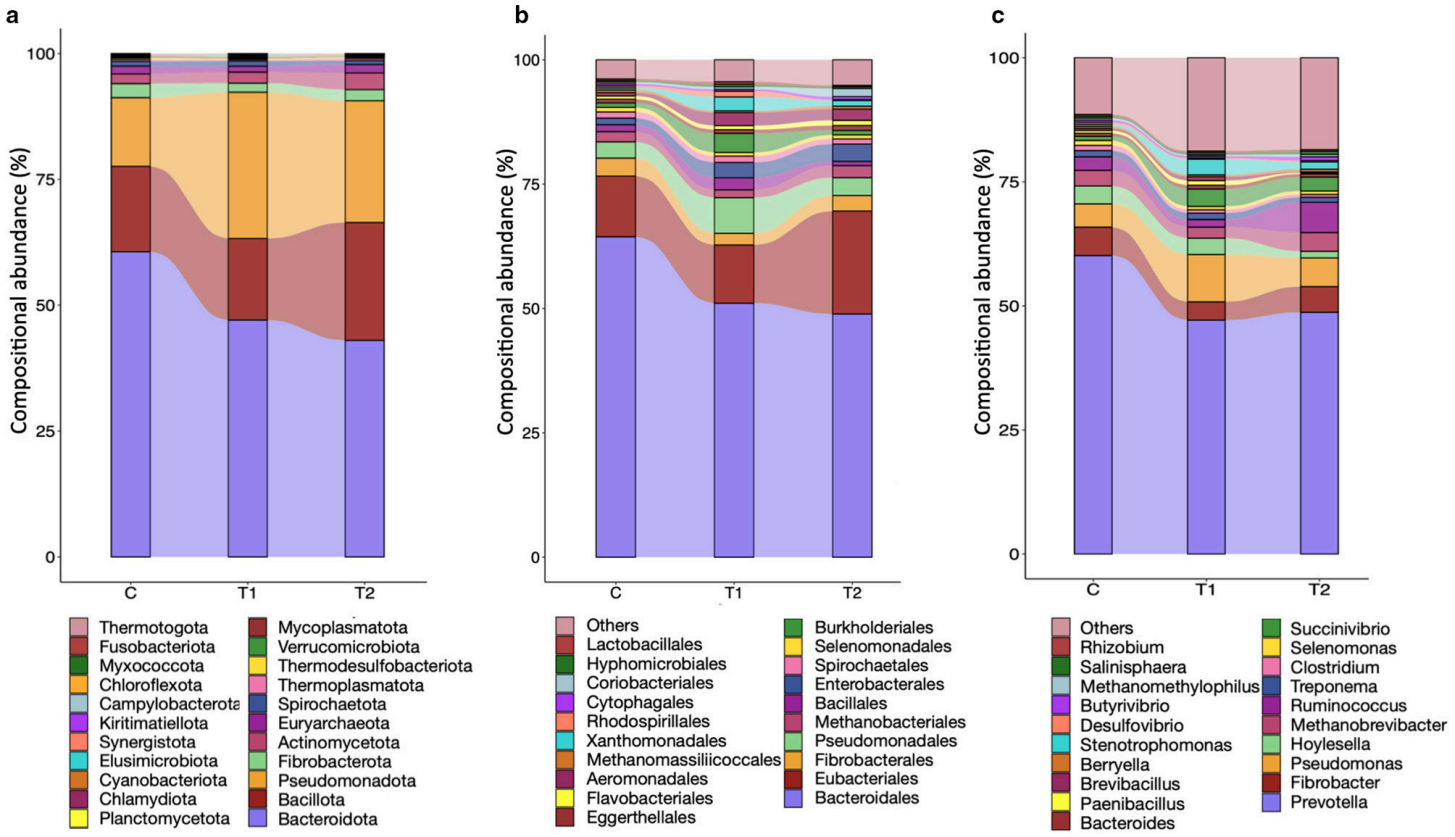
Effect of OBW on the compositional abundances (meta-transcriptome) of rumen microbiota in growing sheep at different taxonomic ranks. **(a)** represents the compositional abundances of microbiota at the phylum level, whereas **(b, c)** represent the abundances at the order and genus levels. C, control group (no replacement of concentrate with oat brewery waste); T_1_, test group 1 where oat brewery waste replaced the equal proportion of concentrate (w/w) and constituted 20% of the total diet; T_2_, test group 2 where oat brewery waste replaced the equal proportion of concentrate (w/w) and constituted 30% of the total diet.

In the study, across the groups, a total of 22 microbial phyla were identified (Supplementary File 1), where Bacteroidota, Bacillota, and Pseudomonadota were the largest microbial phyla, constituting 90% of the ruminal microbiota (Figure 6a). Among these, the Bacteroidota was the most abundant phylum, constituting more than 40% of the total rumen microbiota. The compositional abundance of Bacteroidota was significantly (*P* < 0.05) decreased with the graded replacement of concentrate with OBW in the T_1_ and T_2_ groups. On the contrary, the compositional abundance of the third largest phylum, Pseudomonadota, was increased (*P* < 0.05) in groups T_1_ and T_2_ as compared to group C, whereas the abundance of Bacillota was comparable (*P* > 0.05) among the groups. Similarly, the abundances of other phyla, including Euryarchaeota, were not different among the groups.

At the order level, a total of 101 microbial orders were identified, where microbes affiliated with Bacteroidales constituted ~50% of the ruminal microbiota (Figure 6b). The Eubacteriales was the second largest order of the ruminal microbes; however, their abundance was approximately 3–4 times less than the Bacteroidales. The compositional abundance of Coriobacteriales in the T_2_ group was significantly (*P* > 0.05) greater than the control group, whereas there was no difference in the abundance between groups C and T_1_. The abundance of Synechococcales was increased (*P* < 0.05) with the graded replacement of concentrate with OBW in groups T_1_ and T_2_. Methanogens affiliated with the order Methanobacteriales constituted the largest phylum of the archaea; however, their abundances were not different (*P* > 0.05) among the groups.

A total of 344 microbial genera were present in the rumen. Among these *genera, Prevotella* was the largest, constituting approximately 50% of the total rumen microbiota. *Fibrobacter* and *Pseudomonas* were second and third most abundant microbial genera. However, the abundances of the three largest genera were similar among the groups (Figure 6c). Among the archaea, *Methanobrevibacter* was the most abundant genus, which constituted 2.5%−3.0% of the total microbiota (Figure 6c); nevertheless, their abundances were also not different (*P* > 0.05) among the groups. Replacing concentrate with OBW significantly increased the abundances of *Olsenella, Pseudobutyrivibrio, Marvinbryantia* and *Parafannyhessea*. Conversely, the OBW in the test groups led to a decrease in the abundances of *Eubacterium and Thermoclostridium*.

After removal of host contamination and rRNA, on an average 9,642,117 reads of mRNA per sample were retained from each metatranscriptome library (Supplementary File 1). After *de novo* assembly of mRNA reads, it generated an average of 69,789 contigs (N50 length 862 ± 59 bp). On an average 56.3% ± 2.0% and 30.4% ± 1.2% of mRNA was mapped to the COG and KEGG reference annotated genes.

**Figure 7:**
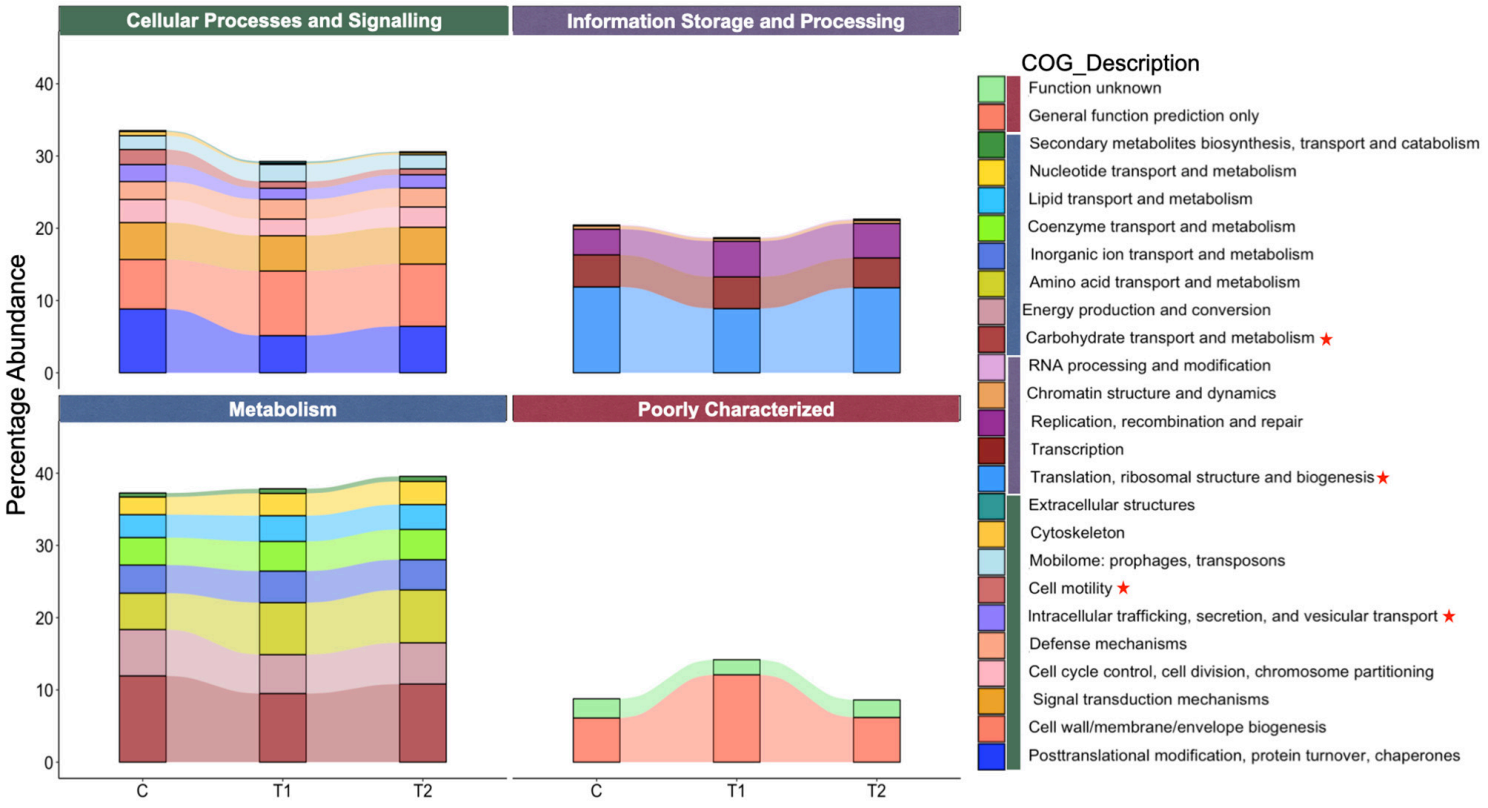
Functional composition and differential analysis of rumen microbiota in growing sheep fed on OBW based diet. The percentage abundance for COG function classification analysis was calculated using COG classifier. The differential abundance for COG function was analyzed by DESeq2 and the COG function categories with significant difference between the groups is marked with red asterik. Different categories of COG functions are represented by four different vertical color bands in the ligand. Green color band represents the cellular processes and signaling, purple band represents information, storage and processing, blue color band represents metabolism, whereas red color band represents the poorly characterized category of COG. C, control group (no replacement of concentrate with oat brewery waste); T_1_, test group 1 where oat brewery waste was replaced the equal proportion of concentrate (w/w) and constituted 20% of the total diet; T_2_, test group 2 where oat brewery waste replaced the equal proportion of concentrate (w/w) and constituted 30% of the total diet.

About 33.5%, 29.3% and 30.6% of non-redundant genes of the total identified COG were assigned to the function cellular processes and signaling in groups C, T_1_, and T_2_, respectively (Figure 7). In cellular processes and signaling category of COG functions, the cell motility and intracellular trafficking, secretion and vascular transport were significantly different between the groups. The cell motility was significantly decreased in T_1_ (*P* = 0.04) and T_2_ (*P* = 0.01) groups as compared to C. Similarly, there was a decrease in intracellular trafficking, secretion and vascular transport in T_1_ (*P* = 0.02) as compared to C, whereas no significant difference (*P* = 0.07) was recorded between C and T_2_. The translation, ribosomal structure and biogenesis function under information storage and processing category of COG was different between the groups (Figure 7). A significant decrease (*P* = 0.04) was recorded between the groups C and T_1_. Similarly, there was a significant difference (*P* = 0.005) in the carbohydrate transport and metabolism function between the C and T_1_ groups. Other COG functions were not found significantly different between the groups.

**Figure 8:**
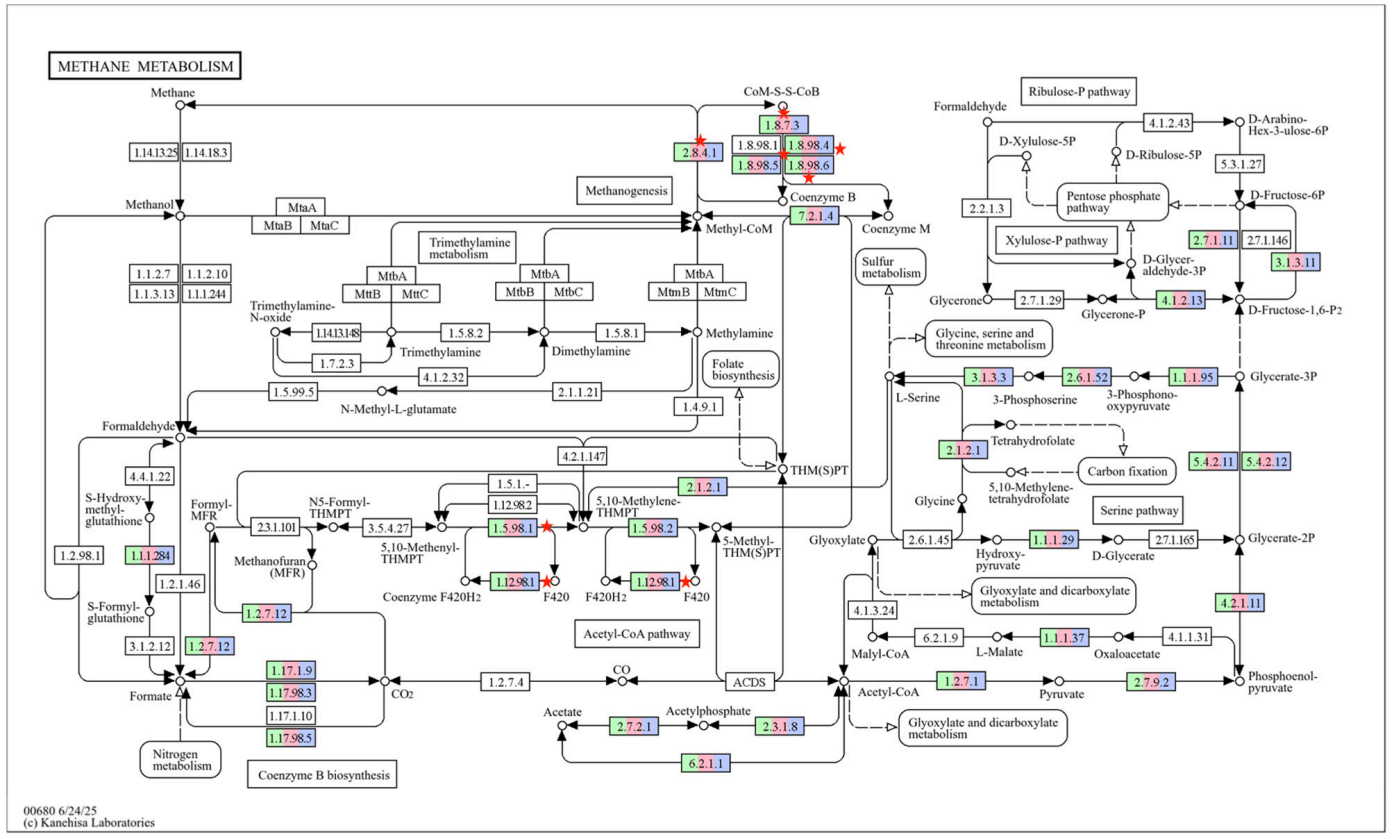
KEGG methane metabolism pathway with EC gene numbers in different groups highlighted in three colors. The green color represents group C, whereas pink and purple colors represent the group T_1_ and T_2_, respectively. The differential abundance for KEGG methane metabolism function was analyzed using DESeq2 and the significant difference in the EC is marked with red asterik. C, control group (no replacement of concentrate with oat brewery waste); T_1_, test group 1 where oat brewery waste was replaced the equal proportion of concentrate (w/w) and constituted 20% of the total diet; T_2_, test group 2 where oat brewery waste replaced the equal proportion of concentrate (w/w) and constituted 30% of the total diet.

KEGG methane metabolism pathway (Figure 8) analysis revealed a significant difference in the EC1.5.98.1-K00319 (methylenetetrahydromethanopterin dehydrogenase), EC1.12.98.1-K00441 (coenzyme F420 hydrogenase), EC1.8.98.6, EC1.8.98.5, EC1.8.98.4, EC1.8.7.3-K03388 (heterodisulfide reductase subunit A2) and EC2.8.4.1-K00401 (methyl coenzyme M reductase sub unit beta). The expression of methylenetetrahydromethanopterin dehydrogenase was significantly decreased (*P* = 0.03) in T_2_ as compared to C. Similarly, the expression of coenzyme F420 hydrogenase was also significantly lower (*P* = 0.004) in T_2_ as compared to C. However, the expression of heterodisulfide reductase subunit A2 was significantly lower in both the T_1_ and T_2_ groups as compared to C. The expression of methyl coenzyme M reductase sub unit beta was lower (*P* = 0.03) in T_2_ as compared to C.

**Figure 9:**
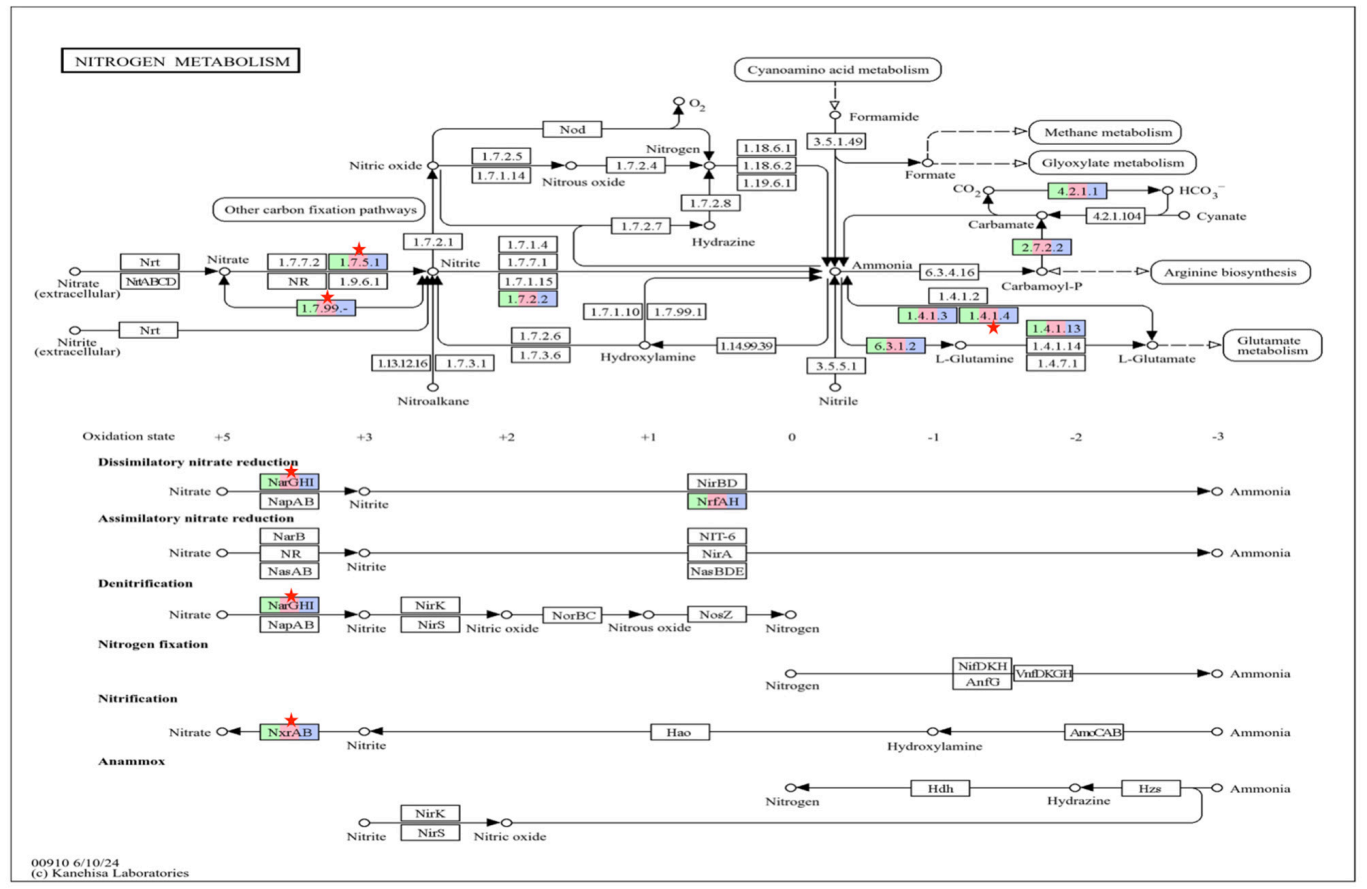
KEGG nitrogen metabolism pathway with EC gene numbers and enzymes in different groups highlighted in three colors. The green color represents group C, whereas pink and purple colors represent the group T_1_ and T_2_, respectively. The differential abundance for KEGG nitrogen metabolism function was analyzed using DESeq2 and the significant difference in the EC is marked with red asterik. C, control group (no replacement of concentrate with oat brewery waste); T_1_, test group 1 where oat brewery waste was replaced the equal proportion of concentrate (w/w) and constituted 20% of the total diet; T_2_, test group 2 where oat brewery waste replaced the equal proportion of concentrate (w/w) and constituted 30% of the total diet.

KEGG nitrogen metabolism pathway (Figure 9) analysis revealed a significant difference in the EC1.1.7.5.2 and EC1.7.99 assigned to K00370 (nitrate reductase) in the present study were expressed significantly lower in T_1_ (*P* = 0.01) and T_2_ (*P* = 0.03) groups as compared to C. Another EC which was significantly different in the present study was 1.4.1.4 assigned to K00262 (glutamate dehydrogenase) was significantly lower in both the test groups T_1_ (*P* = 0.04) and T_2_ (*P* = 0.04) as compared to C.

## Discussion

The search for the farmers' friendly, effective CH4 mitigation strategies is perpetual, with an aim of decreasing enteric CH4 emission using locally available feed resources. The emphasis is on the search for locally available resources. These measures will enhance adoption of the CH4 mitigation technologies in the field by cutting down the cost involved in transportation from one region to another. OBW is an excellent source of protein and energy, nevertheless, due to the high prevalence of fungal infestation can pose a threat to the environment if not disposed of properly. The utilization of fresh OBW in animal feeding will not only tackle the CH4 emissions but will also bridge the gap between feed requirement and availability. An estimate (Gorti et al., 2012) revealed a deficit of 35% and 34% in crude protein and total digestible nutrients, respectively, against the requirement of livestock (20^th^ Livestock Census, GOI, 2019). However, the chemical composition data from the present study established that the fresh OBW, due to higher fiber fractions, cannot replace the concentrate fully in the animal diet. The higher fiber fractions in the OBW-based diet led to a depression in intake and digestibility as compared to the sheep fed on a concentrate-based diet without OBW. This effect was more pronounced in group T_2_, where OBW constituted 30% of the diet; however, at the lower level of replacement (20% T_1_), the difference in nutrient intake between the C and T_1_ groups was not significant, indicating that the moderate level of replacement does not negatively affect nutrient intake.

The correlation between the intake and daily CH4 emission is well documented (Blaxter and Clapperton, 1965; Trupa et al., 2015; Gaviria-Uribe et al., 2020). The significantly lower intake and digestibility in T_2_ as compared to the C and T_1_ groups probably led to the significant reduction in daily CH4 emissions (g/day) in sheep. Comparison per 100 g of DM and OM intake revealed comparable CH4 emissions between control and T_2_ groups. However, CH4 emissions in the T_1_ group, despite the non-significant difference in intake and digestibility, were significantly less than those in the control group, indicating that the reduction in CH4 emissions in the present study was not due to the differences in intake and nutrient digestibility. Further investigation established that in addition to the intake and digestibility, the type of substrate fermented had a more pronounced effect on CH4, as the emission in T_2_ as compared to C and T_1_ per 100 g of digestible DM and OM was significantly higher. The greater concentrations of fiber fractions NDF (4.2%−9.6%) and ADF (2.6%−2.9%) in T_2_ as compared to other groups led to higher CH4 emissions per 100 g of digestible DM and OM. These findings clearly demonstrated that CH4 emissions were indeed dependent on the type of substrate fermented. The earlier studies (Hatew et al., 2015; Yi et al., 2023) corroborated our results, confirming that the fermentation of dietary fiber as compared to starch leads to higher CH4 emissions. The reduction in daily CH4 emissions in the present study at the 20 and 30% OBW in the diet was quite less than that reported previously in an *in vitro* study (Malik et al., 2024). This difference between the studies can be majorly attributed to the compositional difference in the diet composition and the system of evaluation. The diet in this study was composed of 50% concentrate and the remaining 50% concentrate either with or without OBW, whereas the diet in the previous *in vitro* study consisted only of concentrate with or without OBW. The *in vivo* system is a dynamic one, while the *in vitro* evaluation is being done in a closed assembly where end products of the fermentation accumulate until the fermentation is terminated (Malik et al., 2017).

Although the T_2_ group showed a significant decrease in intake and digestibility compared to the C and T_1_ groups, there were no differences in overall growth over 90 days or ADG among the groups. In fact, there seemed to be a pattern of faster growth (gain per kilogram of dry matter intake) when the concentrate was replaced with 20 and 30% of OBW in the diet. The similar ADG in the T_2_ group, even with lower intake and digestibility, might be because it had more CP (12.9% compared to 14.5%) and a higher amount of undegradable protein (UDP) and EE from OBW. The study revealed a big difference in the UDP fraction between the concentrate and OBW (Supplementary Figure S1). As a result, the diet in the T_2_ group contained considerably higher UDP fractions as compared to sheep in the control group. Our results on the high UDP fraction are corroborated by Chiou et al. (1998), who stated that the brewery grain contained a higher fraction of the undegradable protein. The study demonstrated that despite the decreased ruminal fermentation of the diet, the growth rate of the sheep remains unaffected. These findings are consistent with the previous reports (Newbold and Rust, 1990; Kumar et al., 2005; Thakur et al., 2024) that concluded that a high UDP level in the diet increased the conversion efficiency. The significantly higher loss of energy in feces and urine in T_2_ group as compared to control led to a significant decrease in the ME availability in sheep. Apart from the increased loss of energy in the feces and urine, the expenditure of energy by the rumen microbiota for degrading the additional fiber supplied through OBW in the diet probably was more, which leads to the overall less ME availability.

Feeding of brewery waste due to the phenolic compounds can have detrimental effect on the rumen protozoa (Barbosa-Pereira et al., 2014) and that is why the rumen protozoal numbers were significantly decreased in group T_2_ where oat brewery waste constituted 30% of the diet. Though the phenolic content was not determined in the present study; nevertheless, the brewery waste is well known as potential source of phenolic compounds (Barbosa-Pereira et al., 2014). The reduction in ruminal protozoa numbers is another mechanism by which CH4 emissions can be decreased (Malik and Singhal, 2008; Malik et al., 2009; Malik and Singhal, 2016). Protozoa in the rumen live in close proximity to prokaryotic microbes and constitute half of the biomass (Newbold et al., 2015). Protozoa are not essential for animal survival and negatively impact the energetic efficiency of the rumen ecosystem. The obligatory involvement of protozoa in the transfer of H_2_ to the methanogens (Li et al., 2018) keeps the rumen ecosystem working, which otherwise would lead to the inhibition of rumen fermentation. It has been shown that about one-third of CH4 emissions are directly influenced by the rumen protozoa, so the number of these protozoa in the rumen likely affects the CH4 emissions as well. Our results established that a significant reduction in total protozoa, *Entodinimorphs*, and *Holotrichs* in the T_2_ group led to the reduction in enteric CH4 emissions. The similar daily enteric CH4 emissions between the T_1_ and T_2_ groups are due to having about the same number of total protozoa and *Entodinimorphs*, which is why adding more OBW beyond 20% did not further lower CH4 emissions.

The noteworthy reduction in TVFA production demonstrated the adverse effect of replacing concentrate with OBW at the maximum level of 30% of the diet. Nonetheless, replacement at 20% level did not result in any significant reduction in TVFA production. These results indicated that replacing concentrate with OBW at 20% level in the diet was effective in lowering daily enteric CH4 emissions significantly, while not adversely impacting the diet fermentation. Furthermore, the additional replacement of concentrate with OBW at 30% level in the diet negatively impacted the fermentation process without achieving any further reduction in CH4 emissions. As compared to C, the significant reduction in acetate and propionate production in T_2_ group indicated that the inclusion of OBW replacing concentrate and constituted 30% of diet adversely affected the overall fermentation and the effect was more pronounced on acetate and propionate as these are the principal VFA, together constitute more than 90% of the total (Bergman, 1990). This decrease in acetate and propionate production can be attributed to the decreasing ratio of concentrate in the diet (Kljak et al., 2017). The decrease in ammonia-N levels in the OBW groups T_1_ and T_2_ can be associated with reduced ruminal protein degradation, primarily due to the high content of undegradable protein.

The prevalence of Bacteroidota in the rumen microbiota aligns well with earlier findings (Nathani et al., 2015; Wang et al., 2020; Malik et al., 2023, 2024). Nonetheless, the notable reduction in the abundances of Bacteroidota observed in the current study stands in contrast to our earlier findings (Malik et al., 2024), which indicated that replacing concentrate with OBW in the diet had no effect. This variation could result from the differing approaches used for assessing microbial diversity. In the current investigation, microbial abundances were evaluated using metatranscriptomics, while our earlier work relied on a metagenomic approach. Metatranscriptomics is considered to provide a more precise representation of microbial activity than metagenomics, as the latter captures the complete genetic content, including inactive DNA, while the former focuses exclusively on the RNA transcripts of actively expressed genes (Jovel et al., 2022; Malik et al., 2023). The reduced ruminal fermentation observed in the diet with a higher level of OBW in the current study can be associated with the lower abundances of Bacteroidota, which play a key role in the breakdown of complex plant carbohydrates in the rumen (Thomas et al., 2011). In contrast to the Bacteroidota, the higher levels of Pseudomonadota observed in the test groups might be linked to decreased microbial protein synthesis or alterations in the rumen fermentation pattern (Liu et al., 2023). The reduced presence of Pseudomonadota in the current study may be attributed to lower microbial protein synthesis, as OBW had a relatively higher proportion of UDP.

Euryarchaeota stands next to the Bacteroidota, Bacillota, Pseudomonadota, Fibrobacterota, and Actinomycetota in compositional abundance in the rumen. The abundances of the Euryarchaeota align with earlier findings (Malik et al., 2023, 2024, 2025). The comparable levels of methanogens among the various groups at different taxonomic ranks indicated that the gradual incorporation of OBW replacing concentrate in equal proportions ruled out any direct influence on enteric CH4 emissions. The main reason behind the minor changes in the overall rumen microbiota composition between the control and OBW groups could be the inherent resilience mechanism due to the microbiota cross feeding networks, stable ecological niches and buffer against the temporary perturbations during short term feeding of test source for 90 days.

Despite the similar compositional abundances of archaea, the mRNA based functional annotation via KEGG pathway analysis revealed a significant decrease in the expression of various enzymes that led to the production of methane via methylotrpophic or hydrogenotrophic pathways. The significant reduction of methane emission can be attributed to the significantly lower expression of heterodisulfide reductase subunit A2 in T_1_ and T_2_ groups as compared to C. These findings revealed that the feeding of OBW at the selected levels of 20 and 30% of the diet adversely affect the activity of heterodisulfide reductase and therefore leads to a significant reduction in rumen methanogenesis. Further, the replacement of concentrate with OBW at the highest level of 30% of diet in present study significantly lower the activities of methylotrophic archaea methylenetetrahydromethanopterin dehydrogenase and methyl coenzyme M reductase, which would also have contributed to the reduction in methane emission. Similarly, the OBW at the highest level also tend to affect the hydrogenotrophic conenzyme F420 hydrogenase. Despite a non-significant change in the archaeal community composition, the replacement of concentrate with OBW at the selected levels for 90 days in growing sheep significantly and largely affected the activities of methylotrophic archaea, which led to a significant reduction in methane emission. Probably, the extending feeding of OBW in long term may leads to the restructuring in the archaeal community composition by overcoming the microbial resilience and allowing the advantageous microbes to proliferate, horizontal gene transfer and eroding microbiome buffering capacity. As the contribution of individual group of archaea such as methylotrophic, hydrogenotrophic and aceticlastic to the rumen methanogenesis is not known, therefore, at this stage it is difficult to establish that what was the exact contribution of depression in feed intake and decrease in methylotrophic methanogens functionality to the overall reduction in enteric methane due to the OBW. These questions need to be answered through further studies. As the sample size for compositional and functional analysis of the rumen microbiota were smaller (five per group), therefore, could limit the statistical power for detecting differential abundance and expression. In future, the *in vivo* study with more animals may overcome the limitation of statistical power for differential expression. Further, multipoint sampling of rumen fluid rather than single sampling over a feeding cycle would have provided greater insights into the compositional and functional dynamics.

Replacement of concentrate with OBW in both the test groups adversely affected the metabolic capabilities of microbes possess glutamate dehydrogenase, which is accountable for the assimilation of ammonia into microbial protein (Pengpeng and Tan, 2013). The activity of glutamate dehydrogenase is primarily depends on the ruminal ammonia concentration and that is why the lower ammonia concentration in group T_1_ and T_2_ as compared to C could be accountable for the lower expression of glutamate dehydrogenase. In the present study, we established that the OBW has substantially higher fraction of undegradable protein as compared to the concentrate and that is why the OBW based diets in T_1_ and T_2_ groups produced comparatively less ammonia in the rumen. The significant decrease in the expression of nitrate reductase in T_1_ and T_2_ groups could be attributed to the high C/N ratio and phenolic content of the OBW.

The absence of aflatoxin B1 in the fresh OBW indicates that it is safe for consumption. In the following 3 days, traces of aflatoxin B1 (0.20–0.45 ppb) were detected; however, the level was significantly below the permissible limit of the mycotoxin (20 ppb) in animal feed (Devegowda et al., 2019). Aflatoxin B1 from feed sources converts into M1 in dairy animals, posing a health risk to humans (Min et al., 2021). Nonetheless, the conversion of B1 to M1 typically ranges from 1 to 6%, dependent upon the milk yield (Applebaum et al., 1982). Since the present study was conducted on growing animals, further investigation into the transformation to M1 in dairy animals is warranted.

The comparable FCR among the groups revealed that the replacement of concentrate with OBW did not affect the productive performance of the sheep. The FCR reported in this study are in good agreement with a previous report in sheep (Pradeep, 2020). The expenditure incurred (INR) for the feeding of growing sheep during 90 days was 8,395, 6,330, and 4,873 in group C, T_1_ and T_2_, respectively. This study demonstrated that the replacement of concentrate with oat brewery waste at 20 and 30% of diet and the gain achieved in the respective groups substantially decreased the cost of feeding in T_1_ and T_2_ groups. For each kg gain in the growing sheep, the dairy farmers will spend less, approximately 312 and 443 INR in group T_1_ and T_2_ as compared to C.

## Conclusions

From the *in vivo* study in sheep, it can be inferred that the replacement of concentrate with oat brewery waste at 20% level in the diet significantly decreased daily enteric CH4 emissions without any adverse impact on the feed intake, digestibility, and growth performance. Though the feeding of oat brewery waste in short-term (90 days) did not restructure the archaeal community composition, nevertheless it significantly affects the metabolic capabilities of methylotrophic methanogens. Therefore, replacement of concentrate with oat brewery waste at the selected level achieve the reduction in enteric CH4 emissions by altering the enzyme expression of methylotrophic methanogens and decreasing ruminal protozoa without affecting the methanogen community. The farmers will have to spend less towards feeding to achieve the comparable body weight gain and therefore derive monetary benefits. Replacement of concentrate with oat brewery waste beyond specified level did not further reduce daily enteric CH4 emissions, whereas adversely affect the feed intake, nutrient digestibility, fermentation and increased fecal and urine energy loss.

## Data Availability

The data presented in this study are publicly available. The data can be found here: https://www.ncbi.nlm.nih.gov/, accession PRJNA1244113, https://www.ncbi.nlm.nih.gov/sra/?term=PRJNA1244113.

## Supplementary material

Supplementary Figure 1 Rumen degradable (RDP) and undegradable (UDP) fractions of protein in concentrate, oat brewery waste and different groups. C, control group (no replacement of concentrate with oat brewery waste); T_1_, test group 1 where oat brewery waste replaced the equal proportion of concentrate (w/w) and constituted 20% of the total diet; T_2_, test group 2 where oat brewery waste replaced the equal proportion of concentrate (w/w) and constituted 30% of the total diet. Different superscripts on the top of bar represent the significance at a *P* value of 0.05.
