# Supplementary figures and images for: Effect of replacing concentrate diet with oat brewery waste on enteric methane emissions, nutrients intake, digestibility and compositional and functional abundances of rumen microbiota in growing male sheep
Source: Front Microbiol. 2026 Jan 27;17:1646477. doi: 10.3389/fmicb.2026.1646477 (PMC12886344; doi:10.3389/fmicb.2026.1646477)

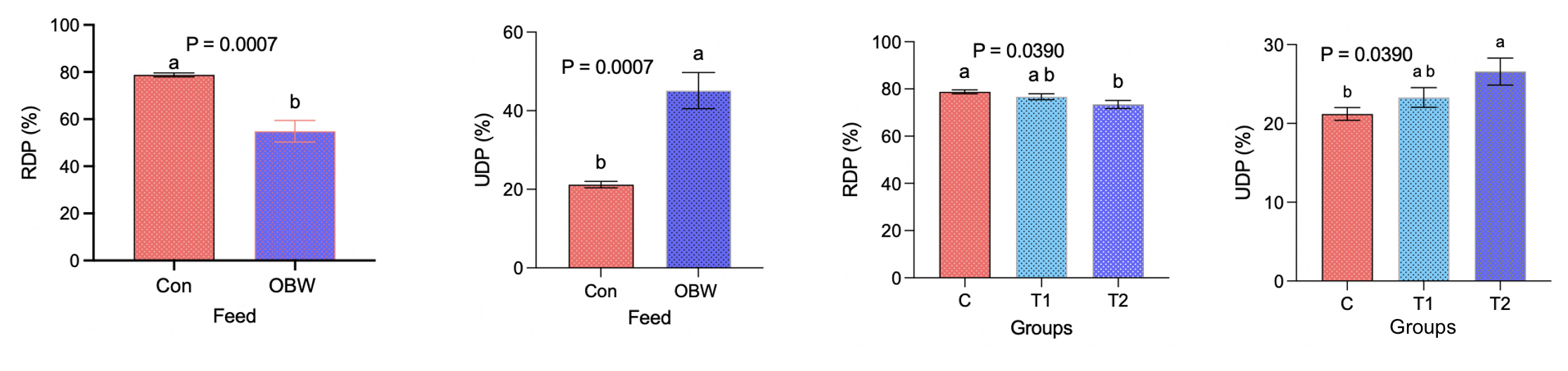

Supplement: Supplementary Figure 1 — Rumen degradable (RDP) and undegradable (UDP) fractions of protein in concentrate, oat brewery waste and different groups. C, control group (no replacement of concentrate with oat brewery waste); T1, test group 1 where oat brewery waste replaced the equal proportion of concentrate (w/w) and constituted 20% of the total diet; T2, test group 2 where oat brewery waste replaced the equal proportion of concentrate (w/w) and constituted 30% of the total diet. Different superscripts on the top of bar represent the significance at a P value of 0.05. [file Image_1.jpeg]
